# Increased left dorsolateral prefrontal cortex density following escitalopram intake during relearning: a randomized, placebo-controlled trial in healthy humans

**DOI:** 10.1177/20451253221132085

**Published:** 2022-11-17

**Authors:** Thomas Vanicek, Murray B. Reed, René Seiger, Godber M. Godbersen, Manfred Klöbl, Jakob Unterholzner, Benjamin Spurny-Dworak, Gregor Gryglewski, Patricia Handschuh, Clemens Schmidt, Christoph Kraus, Thomas Stimpfl, Rainer Rupprecht, Siegfried Kasper, Rupert Lanzenberger

**Affiliations:** Department of Psychiatry and Psychotherapy, Medical University of Vienna, Vienna, Austria; Department of Psychiatry and Psychotherapy, Medical University of Vienna, Vienna, Austria; Department of Psychiatry and Psychotherapy, Medical University of Vienna, Vienna, Austria; Department of Psychiatry and Psychotherapy, Medical University of Vienna, Vienna, Austria; Department of Psychiatry and Psychotherapy, Medical University of Vienna, Vienna, Austria; Department of Psychiatry and Psychotherapy, Medical University of Vienna, Vienna, Austria; Department of Psychiatry and Psychotherapy, Medical University of Vienna, Vienna, Austria; Department of Psychiatry and Psychotherapy, Medical University of Vienna, Vienna, Austria; Department of Psychiatry and Psychotherapy, Medical University of Vienna, Vienna, Austria; Department of Psychiatry and Psychotherapy, Medical University of Vienna, Vienna, Austria; Department of Psychiatry and Psychotherapy, Medical University of Vienna, Vienna, Austria; Clinical Department of Laboratory Medicine, Medical University of Vienna, Vienna, Austria; Department of Psychiatry and Psychotherapy, University of Regensburg, Regensburg, Germany; Department of Molecular Neuroscience, Center for Brain Research, Medical University of Vienna, Vienna, Austria; Department of Psychiatry and Psychotherapy, Medical University of Vienna, Waehringerstr. 18-20, Vienna 1090, Austria

**Keywords:** associative learning, relearning, depression model, escitalopram, neuroplasticity, selective serotonin reuptake inhibitors

## Abstract

**Background:**

Serotonergic agents affect brain plasticity and reverse stress-induced dendritic atrophy in key fronto-limbic brain areas associated with learning and memory.

**Objectives:**

The aim of this study was to investigate effects of the antidepressant escitalopram on gray matter during relearning in healthy individuals to inform a model for depression and the neurobiological processes of recovery.

**Design:**

Randomized double blind placebo control, monocenter study.

**Methods:**

In all, 76 (44 females) healthy individuals performed daily an associative learning task with emotional or non-emotional content over a 3-week period. This was followed by a 3-week relearning period (randomly shuffled association within the content group) with concurrent daily selective serotonin reuptake inhibitor (i.e., 10 mg escitalopram) or placebo intake.

**Results:**

Via voxel-based morphometry and only in individuals that developed sufficient escitalopram blood levels over the 21-day relearing period, an increased density of the left dorsolateral prefrontal cortex was found. When investigating whether there was an interaction between relearning and drug intervention for all participants, regardless of escitalopram levels, no changes in gray matter were detected with either surfaced-based or voxel-based morphometry analyses.

**Conclusion:**

The left dorsolateral prefrontal cortex affects executive function and emotional processing, and is a critical mediator of symptoms and treatment outcomes of depression. In line, the findings suggest that escitalopram facilitates neuroplastic processes in this region if blood levels are sufficient. Contrary to our hypothesis, an effect of escitalopram on brain structure that is dependent of relearning content was not detected. However, this may have been a consequence of the intensity and duration of the interventions.

**Registration:**

ClinicalTrials.gov Identifier: NCT02753738; Trial Name: *Enhancement of learning associated neural plasticity by Selective Serotonin Reuptake Inhibitors*; URL: https://clinicaltrials.gov/ct2/show/NCT02753738.

## Introduction

The brain’s capacity to adapt to a changing environment and effectively navigate daily activities is grounded in neuronal plasticity. Synaptic (re)organization, dendritic remodeling, neuron-glial coupling, and epigenetic processes are mechanisms that underlie neuroplasticity, and consequently facilitate memory formation and consolidation.^1–5^ Brain activity-dependent pre- and postsynaptic specialization peaks in adolescence and continues at significantly lower turnover rates throughout life.^1,2^ Serotonergic modulation has been shown to influence learning and memory across species. Tryptophan depletion studies demonstrated diminished cognitive performance in animals, healthy participants, and patients.^6–8^ In a systematic review of effects of chronic selective serotonin reuptake inhibitors (SSRI) administration in healthy individuals, Knorr *et al.*^9^ reported significant changes in various physiological, behavioral, and neurophysiological parameters, while also depicting marginal to no effects with regards to other parameters. In rodents, SSRIs have been shown to improve memory consolidation and relearning.^10^

The serotonergic neurotransmitter system is involved in neuronal organization and adaptation and affects neuronal circuit formations by regulating neuroplastic processes at the synaptic level.^11^ Serotonergic agents such as SSRIs are frequently prescribed to treat mental disorders such as depression and anxiety^12^ and found to unfold action by modulating cell cascades that are relevant for neuronal restructuring. SSRIs elevate protein synthesis of cyclic adenosine monophosphate (cAMP) response element binding protein (CREB) and brain-derived neurotrophic factor (BDNF), two proteins that affect synaptic formation and subsequently memory and learning.^13–15^ Recent discoveries by Castrén and colleagues indicate a direct link between SSRIs and neuroplasticity that is mediated in part by the affinity of SSRIs to neurotropic growth factor receptors.^16^ Stress models in animals are implemented to explore the relationship between neuropathophysiology and behavior. Such studies have consistently shown an association between dendritic degeneration and a decline in cognitive functioning. In fact, impairments of working memory and attentional set-shifting are consequences of stress.^17,18^ Notably, SSRIs have been shown to reverse both stress-induced hippocampal and prefrontal dendritic atrophy as well as depressive behavior and are also suggested to balance neurotransmission by enhancing the synthesis of synaptic cell adhesion molecules.^19,20^

Structural magnetic resonance imaging (MRI) has been used frequently to examine learning-induced neuroplasticity in numerous interventional studies.^4^ As a consequence of training and learning, physical activity and perceptual stimulation, the expansion of gray matter has been observed in stimulus-specific brain regions, suggesting that high-frequency, non-pharmacological interventions impact neuronal activation and prime MRI-detectable, morphological changes.^21–23^ With regard to brain plasticity, antidepressants have been found to alter gray and white matter as well as neurotransmitter levels.^24–28^ These types of changes have been shown to affect learning and cognition in healthy cohorts, and to be clinically relevant.^9,29^

Several neuroimaging studies have focused on the assessment of SSRIs properties on neural activation during emotional and neutral processing using functional MRI.^26,30,31^ However, surprisingly few studies have addressed structural brain changes following long-term administrations of SSRIs. In two imaging studies, SSRIs were found to affect gray matter of healthy humans and non-human primates, with findings suggesting divergent SSRI effects for healthy compared to depressive individuals.^32,33^ Further investigations are needed, since the mechanisms underlying the serotonergic modulation of gray matter, especially in combination with learning and memory consolidation, are insufficiently understood.

## Aims of the study

This longitudinal drug- and learning-intervention study aimed to test the effects of serotonergic modulation on neuroplasticity using structural MRI during relearning. We hypothesized that SSRIs affect brain areas comprising the hippocampus and parahippocampus during associative learning and relearning, independent of content, whereas relearning with emotional content would expand neuronal recruitment and thus change gray matter of emotion-regulating brain regions such as the amygdala, prefrontal cortex (PFC), insula, and anterior cingulate cortex.

## Methods

### Study design

**Figure 1.**
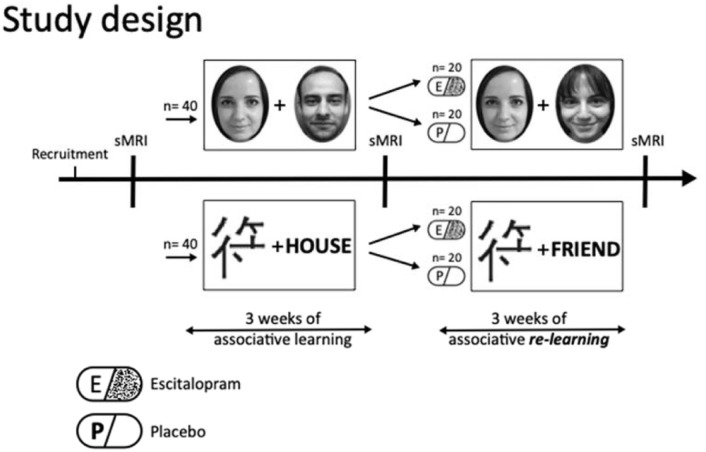
Study design. Healthy participants performed a computer-based association learning paradigm with (face pairs) and without emotional content (Chinese characters and unrelated German nouns) on a daily base for three weeks. Subsequently, individuals had to relearn shuffled associations (within their group) for a further 3 weeks while receiving either a daily dose of 10 mg escitalopram or placebos. MRI measurements were scheduled at baseline, after associational learning (3 weeks) and after associational relearning (another 3 weeks). Study participants were randomly assigned to learning groups and treatment conditions (escitalopram vs placebo). sMRI: structural magnetic resonance imaging.

To assess SSRI-evoked structural brain changes, we conducted a randomized double blind placebo control, monocenter (re)learning study in healthy individuals. Structural MRI measurements were carried out at 3 time-points throughout the study: at baseline, after associative learning and finally after associative relearning, with a respective time-interval of 3 weeks between MRI sessions. Before MRI-assessment and associative learning, participants were randomly assigned to an associative learning group with or without emotional valence as well as to the substance group (escitalopram vs placebo). The emotional group consisted of pairwise matching of face pictures, whereas the group without emotional content comprised of associations of Chinese characters with unrelated German nouns (i.e., the nouns are not accurate and are chosen randomly). The emotional (i.e., faces) group pairs shown indicate positive and neutral valence. After a 3-week learning period, participants either received a daily dose of 10 mg escitalopram or placebo while relearning new associations of the previously learned pairs, within the same group, for a further 3 weeks. For details and illustration, see Figure 1.

### Study participants

The planned sample size of this study was 80 healthy participants that completed the study. General health was assessed through the medical history and a physical examination. A structured clinical interview for *DSM*-IV (SCID I) was conducted to assure mental health. Besides general health, willingness and competence to partake in this study, further inclusion criteria consisted of being between 18 and 55 years of age, right-handed and non-smoking. Exclusion criteria comprised any medical, psychiatric or neurological illness, any lifetime use of SSRIs, first-degree relatives with a history of psychiatric illness, color blindness, non-European ancestry, MRI contraindications and knowledge of the Japanese Kanji or the Chinese Hanzi. Participants that dropped out were replaced.

The study was registered at clinicaltrials.gov with the identifier NCT02753738. The distributions of sex and age in between groups were tested using Fisher’s exact and that of age with a Kruskal–Wallis test.

### Associative learning paradigm

Associative learning paradigms using faces are associated with activation of hippocampal and parahippocampal.^34–36^ Work by our group demonstrated neural activation of the amygdala, the frontal cortex and the fusiform gyrus in individuals performing facial emotion processing paradigms.^37,38^ Also, Chinese characters in combination with German nouns represent complex symbols lacking emotional valence where peak activations in semantic memory related areas was expected. Therefore, each participant was required to learn 200 image pairs via an in-house developed, online platform as previously published.^39,40^ The image pairs were presented sequentially for 5 seconds, whereas each session consisted of a pseudorandom sub-selection (i.e. sampling with replacement) of 52 randomly selected image pairs of either faces or Chinese characters to German nouns. This creates a large subset of different pairs that was presented every day. After 7 days of learning, participants have been familiar with approximately 88% of the whole set by chance. Therefore, 21 days allows for each participant to familiarize themselves with each pair approximately 3 times before the next session MR session. Each learning session was followed by a retrieval phase with a pseudorandom selection of 52 single images from all previously learned pairs. The correct association had to be selected out of 4 possible answers. The faces were extracted from the ‘10k Adult Faces Database’.^41^ Each participant in the faces group was exposed to every facial stimulus during the learning and relearning phases, though at different days due to the randomization. Learning days 1, 22, and 42 were completed during MRI session.

### Study drug administration and monitoring

The verum subgroup received Escitalopram (Cipralex® Lundbeck A/S, provided by the Pharmaceutical Department of the Medical University of Vienna) 10 mg orally per day (o.p.d.) for 21 days during relearning (i.e. after the second MRI). A steady-state blood-level of escitalopram is obtained after 5 to 7 days (~ 5 elimination half-lives) and 10 mg o.p.d. is a therapeutic dose that leads antidepressive effects following 2 to 3 weeks of continuous intake.^12,42^ Also, we previously performed a longitudinal imaging study where escitalopram was administered to patients and healthy individuals and observed a comparably good tolerability.^24,38^ The other half of the study participants received placebo tablets for 21 days as a control group. To warrant adherence to study procedures and in particular to the administration of the study drug, pill count as well as an assessment of escitalopram plasma through levels were preformed 1, 2, and 3 weeks after administration start (i.e. after the second MRI). The last blood sampling was performed directly before the third MRI. Escitalopram plasma levels were assessed with liquid chromatography–tandem mass spectrometry (LC-MS/MS) at the Clinical Department of Laboratory Medicine of the Medical University of Vienna. The therapeutic reference range for escitalopram it is 15–80 ng/ml.^43^

### MRI acquisition and processing

Each MRI session was conducted using a 3 Tesla MR Scanner (MAGNETOM Prisma, Siemens Medical, Erlangen, Germany) and a 64-channel head coil at the High-field MR Center, Medical University of Vienna. Whole brain T1 images were acquired during each MRI session, [Repetition time (TR) = 2300 ms; echo time (TE) = 2.95 ms; inversion time (TI) = 900 ms; flip angle (α) = 9°; PAT = GRAPPA2; matrix = 240 x 256, 176 slices; 1.05 x 1.05 x 1.20 mm^3^; acquisition time (TA) = 5:09 min].

### Surface-based analysis using FreeSurfer 6.0

The automated recon-all pipeline implemented in the FreeSurfer 6·0 software with default parameters was used for cortical surface reconstruction and parcellation of 34 cortical regions for each hemisphere, subcortical regions and the thalamus (Harvard Medical School, Boston, USA; http://surfer.nmr.mgh.harvard.edu/). A within-participant template was created via inverse consistent registration using all time points for the longitudinal processing pipeline, which included the following processing steps: skull stripping, Talairach registration, and initialization of cortical surface reconstruction, cortical atlas registration, and subcortical parcellation.^44^ Cortical thickness and subcortical volumes for the Desikan-Killiany (DK) atlas^45^ were extracted at each time point. After the automated processing, all volumes were visually inspected.

### Voxel-based morphometry (VBM) analysis using CAT12

To calculate the voxel-based morphometric changes, data were processed using the CAT12 toolbox (https://neuro-jena.github.io/cat/) in MATLAB (version 9.4) via the CAT12 longitudinal pipeline with default settings. Prior to preprocessing, all raw data were visually inspected for potential artifacts. For each participant, the scans for all 3 time points were registered, resampled and bias-corrected. Each scan was then skull stripped and segmented into gray matter, white matter and cerebrospinal fluid. Finally, these maps were transformed into MNI space and spatially smoothed using an 8-mm Gaussian kernel. Finally, the CAT12 reports and output data were visually controlled for miss registrations and segmentation errors.

### Statistics

The effects of substance, learning content and time on cortical thickness and subcortical volumes were analyzed using SPSS 25.0. To this end, a four-way repeated measures analyses of variance (rmANOVAs) was set up to test for substance * content * time * region of interest (ROI) interaction effects and lower. According to our hypothesis, we included cortical thickness of the following regions: hippocampus, amygdala, rostral, and caudal anterior cingulate (frontal), parahippocampal, rostral middle frontal (frontal cortex), pars orbitalis (frontal cortex), and the frontal pole. Subsequent, a second rmANOVA was performed including all 34 cortical, subcortical regions, and the thalamus extracted from the DK atlas.^45^ Statistical analysis of the VBM data was performed using the CAT12 toolbox within SPM12 (https://www.fil.ion.ucl.ac.uk/spm/). To elucidate substance x content x time interaction effects on gray matter density on a voxel level a 2 * 2 * 3 rmANOVA was modeled. VBM analyses were corrected for multiple testing using Gaussian random field theory on a cluster level only as implemented in SPM12 where the threshold for significance was set at *p* ⩽ 0.001 uncorrected and subsequently at *p* ⩽ 0.05 family-wise error (FWE)-corrected. The threshold for significance was set at *p* ⩽ 0.05 family-wise error (FWE)-corrected after *p* ⩽ 0.001 uncorrected at the voxel-level. Interaction effects were dropped in case of non-significance in both surface- and volume-based analyses. Thereafter, we repeated rmANOVA only with study participants that developed escitalopram blood levels within the reference range and compared them to age- and sex-matched controls from the placebo group. Also, the mean VBM values of participants in the verum group, extracted from each ROI was correlated with their escitalopram blood plasma levels using Spearman’s correlation. To test if associative learning and relearning occurred, we compared learning and relearning performance levels from the beginning and the end of each learning period. According to a previous published study of our group,^46^ we assessed performance of the initial and final retrieval (to elude outliers due to exceptional sup- or inferior retrieval performance on a single day, we averaged retrieval performance from 3 initial and final days of each period; for details see).^46^ Linear mixed models were estimated for each learning phase separately to test for main and interaction effects, with time points, learning group and substance group as the fixed effects and subject as the random effect (age and sex were included as covariates). Also, to control for learning effects, we compared performance levels (correct answers, mean value of 21 days in %)^47^ of individuals developing proper escitalopram concentrations with individuals beneath the therapeutic range and correlated performance levels with gray matter density of the left DLPFC in participants with proper escitalopram concentrations. The significance threshold was set at *p* ⩽ 0.05. Bonferroni correction was used to correct for all post hoc comparisons in the VBM analysis and for the number of ROIs and post hoc tests for the FreeSurfer parcellation. To correct for different brain sizes and volumes the total intracranial volume was included as a covariate.

## Results

### Study population

Out of the 138 participants recruited only 84 successfully completed both phases of the study. Of the 84 participants, 4 were dropped due to insufficient data quality, further 3 individuals had to be excluded as their escitalopram plasma levels were under the measurable threshold (< 10 ng/mL) and 1 was dropped due to irregular learning performance. For the analyses, 76 participants were encompassed the final sample which included, 44 women, 32 men, with a mean age (±SD) of 25.6 ± 5 years (age range: 18-47 years; *n* = 8 > 30 years of age). No significant group differences regarding age, sex or group participation numbers (*p* > 0.15) were found. See Table 1 for detailed demographics and participant stratification.

**Table 1.** Demographics for all participants included in the final analysis. (A) indicates the demographics of the 1^st^ 3-week learning period, where participants were assigned to either learn faces pairs (emotional content) or Chinese characters and German nouns (non-emotional content). (B) shows the demographics for the second period, where participants had to relearn previously learnt associations (i.e. randomly shuffled pairs) while taking 10 mg/day escitalopram or placebos.

(A)	Faces				Character			
	Mean		SD		Mean		SD	
N	38				38			
Age [years]	25.61		5.40		25.39		4.48	
Sex [M/F]	13/25				19/19			
(B)	Verum		Placebo		Verum		Placebo	
	Mean	SD	Mean	SD	Mean	SD	Mean	SD
N	16		22		19		19	
Age [years]	25.50	5.21	25.91		25.53	4.94	25.26	4.10
Esitalopram plasma concentration [ng/mL]	18.01	10.51	0	0	18.97	9.28	0	0
Sex [M/F]	7/9		6/16		8/11		11/8	

### Side effects assessment of escitalopram and placebos

Thirty-six from 76 healthy participants that completed the relearning period with the last and third MRI developed side effects. None of them developed serious adverse events. Twenty-four out of 33 participants developed mild to moderate side effects (72%) in the drug intervention group, in the placebo group 12 out of 43 (28%). Thirteen out of 17 participants (76%) that developed sufficient escitalopram plasma levels at the third MRI suffered from mild side effects. Similar side effect rates (11/16; 69%) were found in the escitalopram group that did not develop escitalopram plasma concentrations within the reference range. The type side effects did not substantially differ between drug-groups, except for sexual dysregulation (escitalpram-group: 7/33; placebo-group: 0/43). Side effects were mostly present in the first 7 days and did regress thereafter.

### Associative learning and relearning

Linear mixed models were calculated to assess learning and subsequent relearning performances for learning and substance groups. We found a main effect of time (*F* = 125.01, *p* < 0.001), but no significant interaction effect of time and learning group (*F* = 0.04; *p* > 0.1) for the learning period, and similarly for the relearning phase (main effect of time *F* = 60.65, *p* < 0.001; no significant interaction effect of time and learning group *F* < 0.01; *p* > 0.1). The average retrieval performance levels at the beginning and end the associative learning period were 57.0% (SD ± 16.1) and 72.2% (SD ± 20.2), and for the relearning 58.2% (SD ± 19.3) and 67.4% (SD ± 23.4). Post hoc analysis revealed that performance levels increased significantly during the associative learning (*p* < 0.001) and relearning period (*p* < 0.001). The learning group did not have a significant influence on these findings, since the performance levels both learning group did significantly increase with time (for each comparisons *p* < 0.001).

Next, when analyzing data from the associative relearning period, we found no significant time-by-substance interaction effect (*F* < 0.03; *p* > 0.1) and no significant 3-way interaction between time, learning group, and substance group (*F* < 1.69; *p* > 0.1), indicating that participants receiving escitalopram did not significantly differ in terms of learning and relearning from participants taking placebo. Similar increases of average performance levels were found for the escitalopram and placebo group (verum group, initial average performance levels: 58.6%, SD ± 17.6, final average performance levels: 67.7%, SD ± 22.7; control group: 57.9%, SD ± 20.8, and 67.1%, SD ± 24.2; all comparisons *p* < 0.001).

### Analysis of cortical thickness

To test if SSRIs effecting the (para)hippocampal structures during associative relearning and emotional content learning increasing the involvement of the amygdala, prefrontal cortex, insular, and the anterior cingulate cortex, a four-way rmANOVA was conducted to analyze interaction effects on the brain regions listed above. We found no significant 4-way or lower interactions in both cortical and subcortical regions (*p*_Bonferroni_ > 0.05).

To test for interaction effects outside of our hypothesis on the remaining cortical and subcortical parcels, further four two-way rmANOVAs were run. No significant interactions (substance, content by time, ROI) in either cortical or subcortical structures were discovered (*p*_Bonferroni_ > 0.05). Also, no interaction effects of substance or content of learning with time were found in participants that developed adequate blood concentrations.

### Analysis of voxel-based gray matter changes

To assess the influence of substance and learning content interactions on cortical gray matter and subcortical volume, a VBM analysis was also conducted. Again, no 3-way (substance, content by time) or lower interactions were found after correction for multiple comparisons *p*_Bonferroni_ > 0.05.

### Analysis of study participants with sufficient escitalopram blood levels

**Figure 2.**
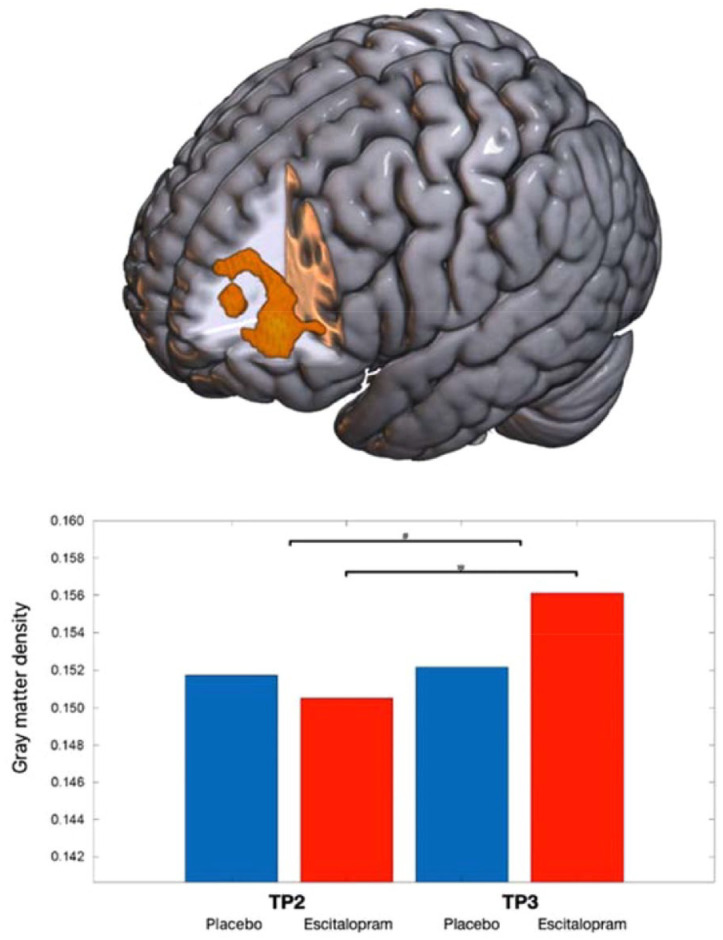
Substance-by-time interaction effect of gray matter density in the left dorsolateral prefrontal cortex. Subsequent to 3 weeks of associative relearning and daily intake of escitalopram 10 mg (orally) or placebos, the verum group showed a significantly greater gray matter density of the left dorsolateral prefrontal cortex (Bonferroni corrected P < 0.05). The upper image depicts a 3D brain and the area in orange the altered area (peak voxel coordination: -42 52-16, x y z). Below: bar graph (red: verum group, blue: placebo group) * depicts significant increases of density of gray matter in the left dorsolateral prefrontal cortex in the escitalopram group over time. # Describes the interaction effect between time-point and substance group. TP, time-point.

**Figure 3.**
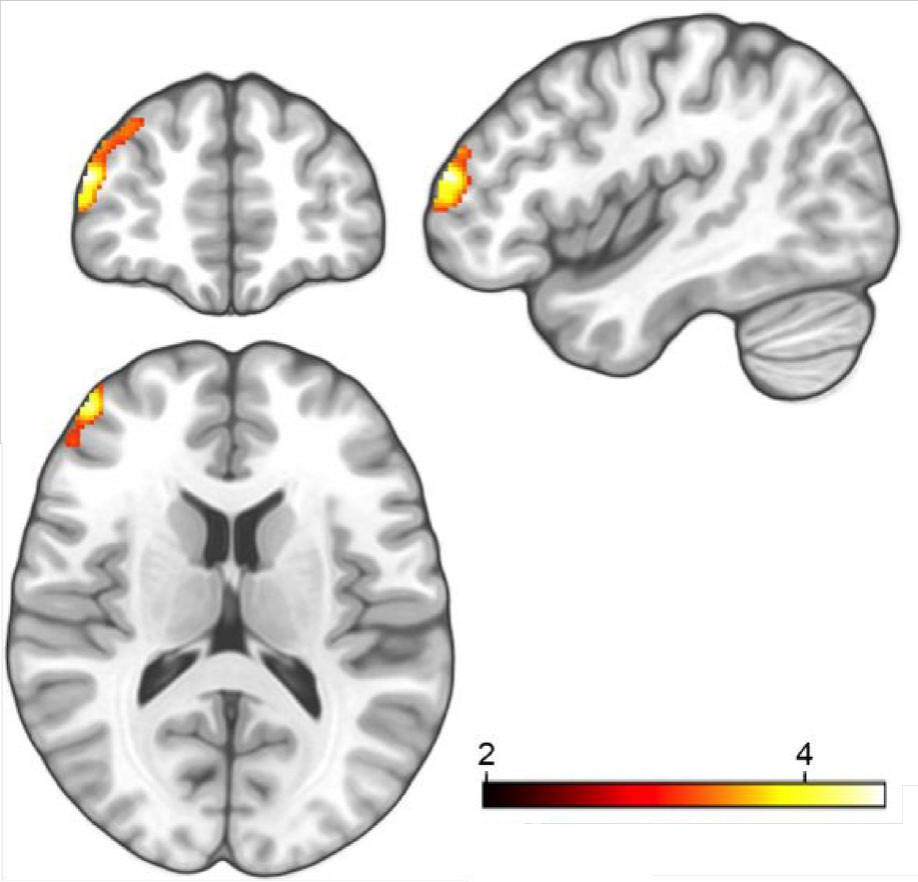
Triplanar view of the significant substance-by-time interaction effect of gray matter density in the left dorsolateral prefrontal cortex. The area in orange depicts one cluster that comprises of 2 sub-clusters (peak voxel coordination: -42 52-16, x y z). The subclusters are interconnected in one area. The color bar represents the degree of the change in density in the left dorsolateral prefrontal cortex after relearning and concomitant study drug intake; orange indicates highest changes.

In study participants that developed escitalopram blood levels within the therapeutic reference range (vs group and sex matched participants of the placebo group) the statistical analysis was repeated (see Table 2 for demographics and escitalopram blood levels). We found no 3-way interaction of substance group x relearning content x time. However, we demonstrate a 2-way interaction between substance (escitalopram vs placebo) x time in this participant group. We examined increased gray matter density in the left PFC subsequent to 3 weeks of escitalopram administration (*p*_Bonferroni_ < 0.05, peak voxel coordination: -42 52-16, x y z; see Figures 2 and 3 for a visual depiction). We found no differences in cortical thickness in left lateral prefrontal regions following 3 weeks of escitalopram administration. A correlation between the mean VBM values of participants that developed sufficient blood levels extracted from the left dorsolateral prefrontal cortex (DLPFC) and the escitalopram blood plasma levels yielded no significant relationship (*p* > 0.1, *r* < 0.2).

**Table 2.** Demographics and escitalopram blood levels of participants that developed escitalopram blood concentrations within the reference range (15-80 ng/mL) after 3 weeks of associative relearning and concomitant escitalopram intake (i.e. 10 mg/day) and of group-, sex-, and age-matched participants that received placebos.

(A)	Faces				Character			
	Mean		SD		Mean		SD	
N	12				22			
Age [years]	25.05		4.64		26.08		3.90	
Sex [M/F]	6/6				10/12			
(B)	Verum		Placebo		Verum		Placebo	
	Mean	SD	Mean	SD	Mean	SD	Mean	SD
N	6		6		11		11	
Age [years]	26.00	4.65	26.17	3.43	24.91	4.72	25.18	4.79
Esitalopram plasma concentration [ng/mL]	28.25	11.30	0	0	23.94	9.42	0	0
Sex [M/F]	3/3		3/3		5/6		5/6	

To control for potential effects of learning performance on the left PFC, we compared performance levels (percentages of correct answers, mean value of 21 days in %, according to Klöbl *et al.*^47^) of participants with escitalopram levels within the therapeutic reference range with participants not developing adequate escitalopram blood concentrations (<15 ng/ml) and found no significant differences between these groups (*p* > 0.05; two-sample t-test). In addition, we correlated performance levels with gray matter density of the left DLPFC in participants with proper escitalopram concentrations. We examined no significant correlation between performance and the left DLPFC (*p* > 0.05; *r* = 0.18).

## Discussion

In this study, we investigated long-term effects of SSRI administration and relearning on gray matter in healthy humans *in vivo.* To ensure methodological reliability, we performed surface-based and voxel-based gray matter analyses. We found no effects of escitalopram (vs placebo) or relearning (as well as on previous learning) on cortical and subcortical gray matter. Among those participants that received escitalopram and developed sufficient drug blood levels (*n* = 17), increased density in the left DLPFC was found. However, in this subgroup, there was no interaction between study drug and relearning content over time and no association of left DLPFC changes with learning parameters.

An abundance of research, from pharmacological to genetic to (pre)clinical studies, has aimed to clarify the role of serotonin in neurobiological mechanisms and its relevance to the development of neuropsychiatric disorder. Given this vast effort in the field of neuroimaging, studies that assess the impact of antidepressants on gray matter *in vivo* using MRI are surprisingly rare. In a placebo-controlled study in non-human primates, 20 mg/kg sertraline (a frequently used SSRI) administration for 18 months (approximately 5 human years) was found to decrease the volume of the hippocampus and anterior cingulate cortex in non-depressed monkeys and increase the hippocampus bilaterally and the left anterior cingulate in depressed monkeys, suggesting opposite trajectories of brain structure formation following sertraline administration.^33^ In a voxel-wise structural study in healthy humans, work by our group shows that escitalopram administered for 10 days prompted gray matter increases in multiple brain regions including the posterior cingulate cortex, ventral precuneus, insula, and fusiform gyrus, and decreases in the pre- and postcentral gyrus.^32^

The hippocampus is the primary brain region responsible for mediating learning and memory, and orchestrates inputs in close relation with other limbic regions and the PFC.^48^ Emotional processes associated with memory and extinction, where fear is mostly utilized as an emotional valence, are mapped to brain regions including the amygdala, orbitofrontal cortex, and hippocampus.^49^ Dysfunctional limbic networks and abnormal monoaminergic signaling are associated with negative affective states, whereas successful treatment with SSRIs has been found to normalize negative attentional bias.^50–52^ We expected to observe escitalopram-associated neuroplastic effects on hippocampal and adjacent structures according to their central involvement in memory and learning and in addition in brain regions related to emotional processing as the amygdala, PFC, insula, and anterior cingulate cortex. Independent of relearning content (i.e. matching face pairs vs Chinese characters to unrelated German nouns), we found no structural changes in the hippocampus and emotion regulation areas subsequent to the learning and relearning period. Results from functional and metabolic MRI studies of our group show escitalopram- and content-specific neuroplastic effects of global functional connectivity during resting state as well as glutamate and GABA levels ^28,47^. Functional connectivity was primarily elevated from the medial PFC to Broca’s area for the emotional condition and bidirectionally between medial PFC and lingual gyrus for the non-emotional condition.^47^ Also, task-related activation during learning demonstrated a significant effect of content, with an amplified activation in the left angular gyrus during the face-matching paradigm compared to the non-emotional condition, which was examined independent of escitalopram administration.^40^ Contrary to our assumption, these learning- and region-specific brain changes did not translate to detectable effects of gray matter.

In a multimodal imaging study that aimed to investigate age-dependent plastic effects in the visual cortex following texture training, white matter changes were found only in the elderly, with apparent functional, but not structural differences observed in the visual cortex after training in younger individuals. The authors suggested that brains of the elderly may require structural change to enhance performance, whereas altered neuronal activation is sufficient to enhance performance in younger people.^53^ Since our study group consisted of healthy individuals who were relatively young (mean 25 years), it is possible that learning interventions altered the brains of individuals at the cell, activity, or functional levels rather than changing brain morphology that is assessed with structural MRI. Another explanation for stable gray matter across the intervention periods could lie within hormone related changes across the menstrual cycle^54–56^, since we randomly included more females than males. Future imaging studies might consider an assessment of menstrual phase, ovarian hormones fluctuations across the menstrual cycle, and even symptoms associated with premenstrual dysphoric disorder.

We included an assessment of pharmacokinetics of escitalopram and its effects on brain regions, where T1 data of individuals that developed blood levels within the therapeutic reference range was analyzed.^43^ In this subsample, escitalopram administration increased the density of the gray matter in the left PFC, particularly in the dorsolateral area. However, no correlation of mean VBM values of the left DLPFC and escitalopram blood levels was found, suggesting that escitalopram has to be present at adequate levels to modulate morphology, but the magnitude of the blood concentration is found to be negligible in our rather small sample for this secondary analysis. The left DLPFC regulates emotional processing and executive control, prioritizing individually relevant information in a context-dependent manner and enhancing goal hierarchy-associated demands^57,58^. In contrast, dysfunction was shown to occur in mood disorders. The attenuation of cerebral blood flow during rest and diminished neuronal activity in the left DLPFC have been reported in patients with depression^59,60^. In a functional MRI study, neuronal activity of the DLPFC was upregulated in depressive patients who were treated with SSRIs over a period of 8 weeks.^61^ Duloxetine, a serotonin and noradrenaline reuptake inhibitor, was found to modulate functional connectivity between the striatum and the left DLPFC, and cognitive behavioral therapy increased functional connectivity between the left DLFPC and the anterior cingulate in depression^61,62^. In addition, brain stimulation methods such as repetitive transcranial magnet stimulation (rTMS) specifically target the left DLPFC in patients with depression, since rTMS-induced activation in this area is described to diminish symptom severity.^63^

Findings from preclinical and clinical investigations focus on elucidating mechanisms of response to SSRIs to provide a basis for the rational of pharmacological treatment for affective disorders. SSRIs act by facilitating various neuroplastic processes,^27^ including binding to the BDNF receptor^16^ and the serotonin transporter on a molecular level ^64^ and modulating functional connectivity networks.^47,65^ As an additional mechanism of action, SSRIs have been found to alter regional cerebral blood flow. Citalopram led to an attenuation of regional cerebral blood flow of the amygdala, fusiform gyrus, insula, and orbitofrontal cortex in healthy individuals.^66^ In patients with depression, escitalopram normalized (i.e. reduced) regional blood flow in the temporal and frontal cortex and in the anterior cingulate cortex,^67^ while a higher baseline cerebral blood flow in the orbitofrontal and anterior cingulate cortex was associated with smaller changes in symptom severity over a 12-week treatment period with sertraline.^68^ Reports from imaging studies support that depression and antidepressive therapy are to some extent related to altered cardiovascular properties as cerebral blood flow, whereas findings are inconclusive across studies. We found no correlation between performance levels and density of the left DLPFC and no interaction between content of relearning and substance group with regards to changes of gray matter. Thus, the presented results challenge the assumption that escitalopram-associated changes in the left DLPFC are merely a consequence of neuroplasticity. However, we observed that participants sufficiently learned and relearned new associations. The specific learning content or if participants received escitalopram or placebo did not have an effect on learning and relearning. We previously demonstrated an effect of escitalopram on relearning performance and on neuronal activation assessed with task-based functional MRI.^40,47^ Also, SSRIs and escitalopram have been described to amplify relearning and induce neuroplasticity in animals and humans ^10,69,70^ Together, these results underline the effects of escitalopram and relearning on neuronal activation, that only partially transfer to changes in brain morphology in our study.

Emotional and cognitive paradigms that effect various aspects of memory provoke neuronal changes in different but also in overlapping brain regions. For instance, the high load of working memory during the performance of both our tasks under escitalopram provides evidence for a more general effect of memory and in particular working memory, independent of other task-specific effects. However, since we did not detect an interaction between substance group, relearning group and structural changes over time, we speculate that the changes the left DLPFC can primarily be explained by escitalopram and the impact of relearning on gray matter is negligible. Within this complex randomized, placebo-controlled 6-week trial, we did not include a group that was excepted from performing daily associated relearning or had to perform a different learning task (e.g. working memory task) to control for primary escitalopram effects, which should be tested in future projects.

Though this study included a rather large sample and utilized state-of-the-art methods, it has limitations that may have affected the interpretation of results. In this study, blood levels of escitalopram were below the reference area of escitalopram in approximately half of the individuals receiving escitalopram considered (*n* = 17/34).^43^ To prevent high dropout rates, individuals received therapeutic doses of escitalopram, since high-dosing is accompanied with higher risks for side effect and thus, study discontinuations.^71^ In a PET study, it was previously shown that even small doses of citalopram are sufficient for occupying the serotonin transporter.^72^ Therefore, escitalopram was assumed to be sufficient for conferring adequate pharmacodynamic drug potency. Nevertheless, we cannot exclude the possibility that an increased dose of escitalopram, or the use of a different type of SSRI may have resulted in sufficient escitalopram blood levels in a greater number of study participants and facilitated substantial differences of gray matter. Also, plasma concentrations of escitalopram were measured at three time points throughout the relearning phase in all participants. However, since the daily drug intake was in the domestic setting, we cannot be certain that all participants adhered precisely to study drug intake procedures. Furthermore, the time interval and intensity of interventions applied in this study may have been sufficient for altering neuronal activity and metabolic factors,^28,73^ despite being insufficient for affecting brain structure. In addition, paradigm parameters such as learning quantity (e.g. time spent on the task, days scheduled) may have also been insufficient to promote morphological changes. Next, although we observed an increase in voxel-based gray matter in the left PFC using the CAT12 toolbox, FreeSufer analysis did not indicate such results. Structural brain studies employing voxel-based and surface-based neuroanatomical techniques are scarce. VBM is one of the most widely used computational neuroanatomy techniques, whereas surface-based analysis gained popularity due to its ability to record subtle neuroanatomical changes in regions of interest. In a structural imaging study in dyslexic patients, voxel-based and surface-based analyses partly revealed coexistent gray matter findings, while in general, more morphological changes were revealed by voxel-based measures.^74^ These findings support the notion that automated voxel-based calculations rely on different variables such as cortical volume and surface area. In addition, to avoid false positives, which were observed in a voxel-based study in which unequal group sizes led to inflated false positive rates,^75^ we matched individuals that received placebos by and learning group (by content), sex and age.

In this randomized placebo-controlled imaging study, we found that 3 weeks of escitalopram administration increased gray matter density in the left DLPFC in individuals that developed sufficient escitalopram blood levels of the drug. Since left DLPFC is involved in neuronal networks underlying depression and represents a key region for treatment outcome, our findings emphasize the left DLPFC as a treatment target for serotonergic agents.

## Declarations

**Ethics approval and consent to participate:** All participants gave written consent to partake in this study and received reimbursement for their participation. The study was approved by the ethics committee of the Medical University of Vienna (EK Nr.: 1739/2016) and was performed in accordance with the Declaration of Helsinki (1964).

**Consent for publication:** Not applicable.

**Availability of data materials:** The data (including supplementary materials) can be made available upon reasonable request to the corresponding author.
